# Practical Monograph on Tapeworm; Illustrated by Two Hundred and Six Cases of the Disease

**Published:** 1844-10

**Authors:** 


### Art. IV.

*Praktische Monographie der Bandwurmkrankheit durch zweihundert sechs Krankheitsfälle erläutert.* Von Dr. Andreas Ignaz Wawruch. Mit einem Vorworte von Dr. Ignaz Rudolf Bischoff.—*Wien*, 1844.

*Practical Monograph on Tapeworm, illustrated by Two Hundred and Six Cases of the Disease.* By Dr. A. I. Wawruch. With a Preface by Dr. J. R. Bischoff.—*Vienna*, 1844. 8vo, pp. 212.

The above work, just issued from the pen of an experienced veteran professor and practitioner of the Austrian school of medicine, upon a curious and somewhat difficult subject, we have perused with considerable satisfaction; and conceive that we shall perform no unprofitable task in furnishing some account of it to our readers.

The author introduces the subject with some interesting gleanings, in the way of history, respecting the extent to which the existence of intestinal worms, with their pathology and treatment, was known to philosophers and physicians of the ancient world, including those of India, Egypt, the Hebrew nation, Greece, and Rome. The perusal of this part of the work will amply repay the German reader, supplying matter to the curious in such matters both instructive and entertaining. It does not, however, fall within our design to furnish any sketch of these literary records; our present object is to exhibit to the reader just so much of the substance of the work as shall appear to possess direct practical importance.

The usual division of tapeworm into the *tænia lata* and *tænia solium* is, in the estimation of our author, quite sufficient for all ordinary considerations; although it is his own conviction that between these two well-marked species there are many intermediate, exercising, however, no differential influence either upon the pathology or treatment.

The physical description and characteristic differences here afforded of the two leading species of tapeworm, correspond very much with those which are given in most elementary treatises on the subject accessible to the English reader; hence, we pass over this portion, and proceed to other matters bearing on the physiology of the subject.

The origin of a worm and the coming into existence of a world, excel, in a like degree, the capacity of the human understanding. The most brilliant and acute theories respecting the mode in which intestinal worms arise have ever crumbled into dust, when the interrogative *how* has been too closely proposed. In Dr. Wawruch's estimation, the problem of spontaneous or equivocal generation must ever remain unsolved; he states, however, that experience speaks too loudly in favour of the independent origin of worms to render it necessary to take refuge in the idea of continuation by generation. The nutrition and growth of the tapeworm, when born, is almost as difficult a matter as the question of its origin; the organs of prehension and absorption being verified with less certainty and precision in this than in many other species. It is left for the departmental student to decide whether the growth of the body be merely from the head downwards, or, what is more probable, by absorption of nutrient fluid through the entire surface of the skin. It is a certain fact that hunger, on the part of the patient, constitutes the most perilous opponent—yea often occasioning the death—of this parasite. Notwithstanding the occasional activity of its movements which, in many instances, distress the patient most grievously, no system of nerves has, as yet, been demonstrated; nor, indeed, any well-marked fibres adapted for motion; the contraction and extension of its joints, as evinced during protrusion of a portion from the rectum, and alternate retraction of the same, leave little doubt, however, of their existence. Moreover, that the movement of the worm through the entire track of the alimentary canal, in each direction, must occur with great activity is clear from the frequency of knots, observable in its track, which can only have been formed by the head having made a sort of leap through loops arranged by the disposition of the body.

Unlike that which obtains in certain other species of worms, the *lumbricus teres* for example, where the sexes occur separately, propagation taking place by the congress of male and female, the tapeworm is hermaphrodite, containing within each individual of the species both sets of organs designed for reproduction. Respecting the precise mode in which this function is performed, a variety of theories have been proposed, none of which, however, are at all matter of demonstration: and, for the present, the question remains entirely undetermined. With respect to the way in which parts torn and cast off are regenerated, the author summarily rejects the old notion, prevalent before the discovery of the head, that the worm has its growth at each extremity; as also the proposition that its increase in length takes place by extension of the individual joints; he himself advances that the tapeworm makes good its length, after fragments are thrown off, through extension of the indistinctly articulated folds about the neck, and by apposition of new joints to the end of the tail; a proposition which he considers in great part proved by Andry's striking experiment, wherein this acute observer stuck a silken thread through a protruded limb; this action, of course, occasioned the animal's speedy withdrawal into the alimentary canal; a month afterwards, the patient took a drastic purge, which caused its expulsion; and, since the experiment, it had obtained an addition of about forty joints below the thread, constituting by so much an increase to its length.

Tænia alone, amongst intestinal worms, would appear occasionally to attain a considerable age; the more so, in the instances where it happens to arise in the more vigorous stage of human existence. There is always a difficulty, however, in determining the actual age, in consequence of the uncertainty, whether the duration of the disease be kept up by the presence of one and the same individual, or by continuous regeneration. In one of his own cases, the author states that the disease had had an existence of thirty-five years; and, notwithstanding the apparently successful application, repeatedly, of what had been considered an effective cure, the patient was yet not freed. The age can with little certainty be predicated from the appearance of the portions passed off. Generally speaking, however, the young one is tender, and readily torn; the adult (so to speak) is firm in texture, vigorous, and vivacious; and the aged has commonly a surface marked by frequent wrinkles, and its body is, moreover, of a leather-like tenacity. Sometimes the worm will grow old with man himself, and accompany him even to the grave.

Tapeworm has its principal seat in the small intestines, although, on account of its length, it may extend through the entire track of the alimentary canal, from the pylorus down to the rectum. The presence of this intestinal pest would seem to be considerably under the influence of certain specialities in the soil; thus, there are countries in which it is seldom or never observed; for example, in Quito, and in the higher tracts of Bohemia; a circumstance explicable in part probably by the great elevation of these places above the level of the sea. Low lands subject to floods, the vicinity of the ocean, especially at the mouths of great rivers, and deep valleys, seem most exposed to this evil. What is somewhat remarkable, particular countries seem to possess a species proper to themselves, and some even a distinct genus. The inhabitant of Switzerland, for instance, whilst in his own country, will suffer from the presence of the *tænia lata;* this shall have been got rid of, and, on changing his residence for Vienna, he shall be infested with one of another genus, the *tænia solium.* The German, emigrating to Russia, shall experience the development of the *tænia lata,* a species rarely if ever occurring in his own country. These are curious phenomena, complicating still more the difficult inquiry respecting the origin of intestinal worms.

As regards the symptoms which denote the existence of this disease, it is with tænia as with others of the entozoa, the only certain indication of its presence is the actual dejection of some part of the parasite, seeing that every morbid feeling or appearance induced by it may be occasioned by other causes. Nevertheless, the intensity and duration of the symptoms, with the mode in which they are associated, will, in a great many instances, furnish the highest probability respecting the true character of the ailment, and sometimes render it all but matter of absolute certainty. Signs of derangement in the state and functions of the alimentary canal, with the sympathetic results, constitute the sum of the symptoms; and of these we have a graphic account in the work before us. As it corresponds, however, very much with the experience and reading of all who have, in any degree, studied this matter, we shall not transcribe any portion of it, but proceed to the examination of the next chapter.

*Diagnosis.* It has been before stated that no combination of suspicious symptoms can render the presence of the tapeworm-disease a matter completely assured; the dejection of some portion of the animal is required to lead to a certain diagnosis; but, generally speaking, a mere glance at the rejected fragment will enable the experienced physician to recognize, not only the fact of its being tapeworm, but, moreover, the particular species to which it belongs. Sometimes the patient's description of the part thrown off will be sufficient for the practitioner to determine whether the disease be the tænia *lata* or *solium;* for Dr. Wawruch states, as a conclusion arrived at from his own observations, that the former parts only with great pieces of at least from six to twelve inches in length; whilst the latter generally casts off a number of little gourd-like limbs. This diagnostic circumstance rests upon the different structure of the two species; the one is rendered firm and coherent in its texture by certain longitudinal lines running down its body, whilst the other, besides being deficient in these lines, possesses a much looser tissue, and one which, during intestinal action, is very readily torn.

As exhibiting the facility of mistake in the diagnosis, on the part even of the most experienced, and the necessity of careful investigation in all cases prior to the exhibition of powerful remedies, we take the following statement:

"I cannot refrain from mentioning a remarkable case which placed me, in making the diagnosis, in the most painful embarrassment, at least for a short time. A patient, who for some years had suffered from worms, besought the aid of the late most excellent Dr. Bremser, (deceased, alas, too early for science!) who, after a brief and superficial examination, prescribed for him his electuary and a small flask of the (so called) Bremserian oil, doubtless upon the supposition that he was infested with tapeworm. After a long and fruitless employment of both medicines, the patient became affected with the most vehement griping, bloody urine, tenesmus, and finally an excessive diarrhea, which caused the ejection of an entire roll of half-decayed material and tape-like skins. In the certain conviction that the destruction of his monster had been accomplished, the patient ran to Dr. Bremser, and related the occurrence, who, trusting to the account, fortified the patient in the glad supposition, and counselled a perseverance in the use of the stinking oil. This was done, punctually, for some time. But the bloody urine, and a vehement burning pain in both kidneys, compelled the patient to desist from taking the oil. The wretched man now came to me, and brought in a glass the portions that had been cast off by stool. At first sight I was startled, and hesitated so about the diagnosis, that I requested the person seeking my advice to leave the problematical fragments with me for further examination. I soon attained complete certainty. I remarked that the long skins lying flatly over one another were unjointed, that indeed they must have been cylindrical; since, by obstructing the air at one end with a small pointed reed, they could be inflated, and be made to assume a rounded tubular form. I saw now, plainly enough, that they were *exuviæ* of the common round worms, burst, and, in great part, decayed." (p. 47.)

It is observed that the diagnosis of tapeworm malady, when no ejection of fragments takes place, is so difficult and uncertain as to be exceeded by that of no other disease. It places, at times, the most experienced practitioner in the greatest embarrassment; seeing that many of its general symptoms are common to it and several other diseases, from which, however, before treatment, it must be carefully distinguished. The author enumerates colic, hysteria, hypochondriasis, hemorrhoidal affections, common intestinal worms, and certain nervous affections, as occasionally presenting features likely to complicate the diagnosis, in so far as they, in common with tapeworm, disturb in a variety of ways the functions of the abdominal viscera.

*Etiology.* The etiology of this disease is discussed under the two heads of "predisposition" and "exciting causes." Among the circumstances *predisposing* to the malady, our author mentions sex as exciting no inconsiderable influence, the female being more liable to its attacks than the male. For this greater tendency to the development of tapeworm on the part of the female, no satisfactory reasons can be given ; it is an observed fact; and the causes are as difficult of explanation as the disproportionate prevalence of gout, apoplexy, or stone in the bladder, with the other sex. Some reason may probably be afforded in the earlier occurrence of puberty in the female, in the softer skin, and in the weaker condition of the digestive organs, and consequent greater abundance of mucus, as tænia is most frequently coincident with the reproductive stage of existence, and general laxity of tissue ; and although it cannot be denied that occasionally, both in old age and at the earliest period, this worm will be found, it is yet a comparatively rare circumstance and an exception to the rule. Dr. Wawruch states that, in all his experience, he had never had to treat a female in whom the first symptoms seemed to have arisen after the age of childbearing. Each epoch of life would appear to favour some peculiar parasitic formation ; thus, infancy tends mainly to the production of round worms, ascarides, and common lice, whilst senility seems possessed of immunity from these annoyances ; and, in correspondence with such facts, it is considered that the origin of tapeworm has some special alliance with the generative period of life, and its more vigorous stages.

Notwithstanding the objections that many have offered to the idea of there being any hereditary predisposition to this affection, it is here maintained that the tendency is frequently propagated from parent to child, just as much as the liability to gout, epilepsy, mania, hysteria, and several other ailments of a like kind. The author does not teach that it is the actual *material of generation* which is transmitted, but rather that it is an unfathomable *something* which no human mind will ever thoroughly appreciate, but a something which may yet be demonstrated as truly existing. An irregular mode of life, interfering with the integrity of the digestive function, would appear to operate at one and the same time, as a predisposing and as an exciting cause; and, for obvious reasons, whether the malady be regarded as mainly dependent upon a cachectic habit of body, or as the consequence of nervous debility. The occupation is a circumstance to be studied, as probably standing in some relation to the development of this disease. Dr. Wawruch, in reviewing his registers of cases, considers that an undue preponderance obtains among shoemakers, tailors, cooks, and butchers; a circumstance probably to be explained by these classes frequenting, in undue proportion, the hospital where the observations were chiefly made. The influence of the probable injury done to the energy of the assimilative powers by a sedentary mode of life in some of these instances, or by excess or deficiency of proper nutrition in others, is duly recognized; but yet, to explain the frequent occurrence of tapeworm among butchers, he suggests, with some confidence, that the emanation from fresh-slaughtered cattle, as a true animal material, not only contributes to obesity, but must also tend to the generation of parasitic organisms; an idea which he leaves to the physiologist and practitioner to estimate at what they regard as its just worth. We think that the fact, so far as true, receives a simpler explanation from the well-known circumstance that these men most commonly overload and unduly stimulate the stomach. Our own experience—not very extensive, however—has not led us to ally the affection especially to any particular business; but we have generally observed it to prevail where causes prejudicial to the assimilative functions have operated to a great extent, irrespective of class or occupation.

Amongst other predisposing circumstances the existence of previous attacks of intermittent fever is here mentioned; and the author states that he brings to this idea, long since advanced, though often disputed, the solid support of facts. He observes that both diseases belong to the most vigorous epoch of life—to that period when the generative function is the most powerful; that intermittent fever almost ever leaves its after-evils in the viscera of the abdomen, the locality of tænia; further, that in those countries where the one affection prevails extensively, so does the other; that, for example, in Prague, where ague is seldom heard of, tapeworm is almost unknown; and that in Vienna the two diseases exist both in large proportion. Finally, he affirms that, upon a revision and comparison of his hospital registers for upwards of twenty years, almost all who were treated for tænia had suffered at an earlier period from intermittent fever; a fact to the confirmation of which, or otherwise, the attention of the profession in this country would be well directed. Fever less affections of the skin—such as the itch, scaly tetter, certain forms of porrigo, and herpes—operate, according to our author, as predisposing causes; a conclusion deduced by himself from his own observations, and confirmed, he informs us, by those of his friend Bischoff; and one attempted to be explained by the intimate relations subsisting between the skin and mucous membrane of the alimentary canal.

In discussing the exciting causes of the disease in question, Dr. Wawruch, at some length, proposes to show what he conceives to be the erroneous character of the views commonly entertained with respect to the influence of diet; he maintains that almost the exclusive adoption of a purely vegetable diet, or of one vegeto-animal—such as eggs, butter, milk, cheese, and so on—is not conducive, as many have supposed, to the generation of worms; and in support of this proposition, he appeals to numerous facts, including his own recorded and detailed experience. He adduces the statement of Professor Reinlein, who had paid considerable attention to this affection, that in a cloister where, for an indefinite period, the rule had been to partake only of vegetable diet, with an occasional use of eggs, milk, butter, cheese, and fish, not one of its inmates had been ever known to suffer from tapeworm. A rich, animal, and stimulating diet he regards as a much more powerful cause of the development of the malady; and, as before stated, his own patients have been persons subjected to the operation of this influence, in very undue proportion. He mentions, moreover, that, on appealing to classical antiquity, a confirmation of this view will be obtained; for Theophrastus of Cresus affirms that in Athens, except among the Athletics, tapeworm was hardly ever witnessed; and the diet of these latter was notoriously of a gross, animal kind. Thus, when Diogenes the cynic was asked why the athletics were so rude and uncouth, he replied, with his customary abruptness, because *they lived on hogs' lard and cows' flesh.* Our author states that the conclusion in this respect has been forced upon him by experience, as it is in opposition to his preconceived opinions. He allows, however, that the following out of any plan of diet, if succeeded by any notable derangement of the alimentary functions may tend, in the predisposed, to originate the parasite. We think so.

*Terminations.* Tapeworm has never been known to issue directly in death, not even in those cases where the patient, deprived of all medical aid, has been affected by it uninterruptedly, from earliest youth to old age. Indeed, it may be affirmed that, in many cases, human life is so little endangered by its presence, that sometimes, in opening a dead body, a large tapeworm will be discovered, of the previous existence of which neither the physician, from observation, nor the patient, from feeling, have had the remotest idea. An actual cure, however, rrom the unaided efforts of nature is so exceedingly rare, that it should never be anticipated in any individual instance; although a spontaneous liberation will occasionally occur, and that, too, without being followed by regeneration. The influence of tænia in the production of other ailments, sympathetic or more remote, has been vehemently maintained and as strenuously opposed. Whilst many names of high repute have held that a malady is hardly to be found, especially affecting the nervous system, that may not be induced by intestinal worms; others, of equal authority, have contended that their presence is conducive to the perfection of health, by their fulfilment of an appropriate office in the alimentary canal—probably the absorption of some viscous residuum of the chyle. It is truly a melancholy task for the student in medical literature to acquaint himself with much of the past history of his profession, discovering, as he almost ever does, the total want of harmony in the results obtained, up to a late period, by observers in so many departments; it is nearly always, as in the present case, one group of authorities say one thing, another say just the opposite of the foregoing, and the truth being seldom altogether with either party. We have read carefully over the chapter in the volume before us, in discussion of the terminations of the tapeworm-disease, and cannot help thinking that, although Dr. Wawruch is at no extreme point, he somewhat overrates the secondary consequences. Our own conclusions —deduced alike from reading and experience—certainly are, that no *special* ills flow from the presence of tænia, but only such as usually result from protracted irritation of the gastro-intestinal mucous membrane, upon whatever cause dependent; and that just in so far as worms of every kind aggravate or induce this state of things in particular individuals, they bring about the diseases of a class generally recognized as coming from this source; and that, in habits of body attended by undue sensibility of the lining tissues of the stomach and bowels, tapeworm may induce slow hectic fever, marasmus, cerebral disease, convulsions, and so on, in the same way as such results will often arise in the absence of all parasitic formations.

*Prognosis.* As before observed, tænia prevails more extensively in the female than in the male sex; and, in like manner, success in treatment is to be anticipated with the latter much more readily than with the former. The explanation of this phenomenon is to be sought in the firmer corporeal structure of the male, and in the greater activity of man's general career. On these accounts the male system allows of a freer employment of the means of cure, and, where necessary, of a more protracted continuance thereof. Another circumstance favouring the prognosis, with respect to the curability of this disease in the male, is to be found in the comparative uniformity of condition that obtains in man. Women, from very slight causes, are liable to fainting, to hemorrhages, to convulsive attacks, and nervous affections of every kind; to say nothing of the occurrence of the catamenial periods, of pregnancy, or parturition, lactation, and the more decided climacteric changes; all which things offer special hindrance, in many instances, to the adoption of appropriate therapeutical measures, or at least to an adequate perseverance in the same.

As the malady under consideration is one mainly affecting the mature period of life, from the accession of puberty to full manhood, and through the whole of middle age, so at those times is the prognosis of cure the most certain. When the disease, however, does occur in children sufficiently energetic measures, with a view to expulsion, can rarely be had recourse to, for obvious reasons, especially in the case of such as are rickety and scrofulous. In like manner, old people but little endure effective treatment, particularly if suffering from habitual giddiness, asthma, chronic cough, debility of the bladder, or threatnings of apoplexy. Our author goes on to speak of the influence, as modifying the prognosis, of temperament, corporeal structure, mode of life, hereditary constitution, idiosyncrasy, and some other circumstances. We do not, in his remarks upon these matters, notice anything very striking; they seem introduced more to give completeness to the discussion than for their novelty, being chiefly a repetition, in other words, of what had been previously advanced, and which we have before alluded to.

The tænia lata is more readily expelled than the tænia solium, and that without there being any difference in the remedies employed. No satisfactory reason can be given for this fact. It is suggested that the former species may be endowed with life less *intensively*, and, on that account, be the more speedily destroyed by pharmaceutical means. The fact itself, however, the author puts in the form of an aphorism, and rests it upon simple experience. In the same way, he advances a series of propositions, constituting what he calls the "empirical prognosis," following each of them up with brief explanatory observations. Some of these we shall translate literally, and there leave them; premising only that in each of the following aphorisms he speaks but of the tænia solium (kettenwurm); though, from the context, we presume that he refers to the tapeworm generally.

The advanced and completely formed tapeworm is more easily expelled than one too young. Joints that are of a decayed, withered, ill-coloured, leathery, and shrivelled appearance indicate a favorable prognosis; but those of a bright white character, and in active motion, point to one that is less so. The tapeworm which has but lately parted with considerable portions of its length is more difficult to expel than that which daily allows individual joints to break off, be these ever so numerous. The tapeworm, frequently but ineffectually attacked by strong remedies defies the expellent treatment (*abtreibekur,* a term apparently used exclusively in reference to his own method,) much longer than that which has for years been uninterfered with. The tapeworm, associated with round worms or with ascarides, is seldom or never expelled in the first course of treatment.

The accession of vomiting some time after the medicines have been taken often indeed delays, but does not always frustrate the successful issue. The occurrence of diarrhea too early or too late is prejudicial to success. The more courageously the patient takes the remedies, and the less unpleasantness he experiences from them, the more certain is a fortunate result. The success of a first attempt at cure guarantees a similar result a second time, in case of a regeneration of the worm. Success occurs more frequently in winter than in summer; greater circumspection in the treatment, however, being required in the winter season. The next aphorism may probably provoke a smile, on which account we forewarn the reader. The last four or five days of the waning moon seem favorable to a happy issue. The author, however, states that he has, for nearly twenty years, been in the habit of watching the real or imaginal relations subsisting between intestinal worms and the phases of the moon; and as a simple affair of experience, which he does not attempt to explain, he avows his reliance upon the accuracy of the proposition just adduced.

The prognosis in respect of the probable regeneration after expulsion is set forth in the following propositions, each, as before, being succeeded in the work itself, by an appropriate commentary. The chief of these we shall give literally. The expulsion of the worm, together with its *capital* extremity, does not always ensure against the reappearance of the same. The inability to discover the thin cervical portion, with the head, is no proof of failure of the treatment, and furnishes no ground for an unfavorable prognosis. It is, thence, only a prejudice, that the tapeworm, if torn, must always be reproduced. The period (after expulsion) of six, eight, at most ten weeks, is to be regarded as the surest criterion of there being no probable regeneration. The expulsion of two or more young tæniæ at the same time gives the least favorable prognosis. The continuance, on the part of a patient, of the previous mode of life, after expulsion, lets the speedy reappearance of the tænia be feared ; and the contrary, in the opposite case. The frequent repetition of the expulsive treatment seems able, with certainty, to extirpate the worm at last.

*Therapeutics.* The curative treatment of tapeworm is one of the most difficult problems in practical medicine ; a proposition, the proof of which is eloquently furnished by the host of remedies recommended and employed for thousands of years. Many practitioners, indeed, have affirmed with some truth that, in some cases, it is easier to kill the man than the worm. In adopting the treatment for expulsion shortly to be described, certain contra-indications are to be borne in mind, which either prohibit its employment altogether, or at least necessitates its postponement; these, for the most part, are included in considerations respecting age, sex, peculiar habit of body, actual or previous disease, with some other circumstances, most of which have been before alluded to. We notice one statement in this chapter, which we have a difficulty in reconciling with another before quoted under the head of prognosis; it is set forth that the cold, wet weather of autumn, and the vigorous cold of winter, are far less favorable to success in treatment, than the mild, serene weather of summer; whereas, a few pages previously, the author had dogmatically announced that success followed treatment more frequently in winter than in summer. The truth very likely is, that temperate weather, as interfering least with the general health, is the most conducive to success; and, it is to be supposed that in the enunciation of the former aphorism, our author contemplated a mild winter in opposition to a high temperature in summer, which latter at all times enfeebles the digestive functions.

The method of treatment proposed by Dr. Wawruch, and pursued by him for twenty years with the happiest results, is exhibited and discussed in three divisions ; 1, the *preparatory* or abstinent treatment; 2, the *curative* treatment proper, and 3, the *after* treatment.

1. In preparing the patient for the administration of remedies destructive to the worm, our author for three or four days previously withdraws all solid food, allowing a little mild soup several times in the day. In order to render the alimentary canal more susceptible to the coming treatment, the patient, during this period, takes the following solvent:

℞ Radicis Cichorei et Taraxaci āā unciam unam coque in sufficiente quantitate aquæ per mediam horam; Colaturæ fortiter expressæ unciarum sex adde
Salis ammoniaci depurati scruplum,
Syrupi Cichorei cum Rheo unciam semis.
Detur usui, signetur: "Two tablespoonfuls to be taken every two hours."

The object in the above prescription is to carry away by gentle means all impurities in the *primæ viæ,* at the same time that a more suitable predisposition is obtained for the next step. When, after a careful consideration of all the possible contra-indications, the time for proceeding becomes fixed, the patient, on the evening previous, must take a rich panado, made of bread-crumb, water, and several ounces of butter; and, at the same time, he must have, within the space of an hour or two, three or four enemata administered, constituted of linseed tea with equal parts of milk and oil. On the following morning, that of the appointed day, there must be a repetition of the same clysters and the same soup.

To this preliminary treatment, Dr. Wawruch attaches a very great importance; so much so, that he ventures to affirm that the success or non-success of the entire plan will often depend upon its strict observance; as the temporary withdrawal of the customary nutrition attacks the vitality of the parasite at once; for, according to an old saying of Alexander of Tralles, " hunger is the tapeworm's mightiest foe." Abstinence from food ever aggravates the subjective symptoms, inducing a sort of desperation on the part of the intestinal lodger; and, after this has continued for some time, its rage for food becomes so considerable that whatever comes in its way is greedily consumed, even the medicament administered for its destruction. Another circumstance explaining the utility of the abstinent treatment consists in the fact that, in this way, the excitability of the alimentary canal becomes exalted, and, under the influence of the appropriate drug, its peristaltic action determines more powerfully downwards, expelling the entire contents of the intestines, inclusive of the worm rolled up. Failure in most cases is to be attributed, when it occurs, to insufficient preliminary fasting; and, indeed, in many of his own recorded cases, the cure was completed by this alone, the tænia being passed without a grain of medicine having been taken for the purpose. The employment of the mucilaginous and oily panado and enemata, whilst it enables the patient to endure the abstinence from other matters, has its use also in protecting him from being too powerfully influenced by the succeeding remedies, which otherwise might excite hyperemesis or hypercatharsis.

2. The measures for bringing about the expulsion of the tapeworm must begin early on the fourth day, just on the completion of the preparatory discipline before mentioned. The means themselves are of a twofold character, having for their object, first, to destroy the worm, and then to expel it from the body. For the first purpose, castor-oil and the male fern, are the remedies recommended; and, in the case of the former, it is of consequence that it should be recent, and have been expressed from the seeds including the husks, in which latter our author says the anthelmintic property seems to reside. He is accustomed to exhibit this medicine according to the following formula:

℞ Olei Ricini Americani ex seminibus cum pelliculis recenter pressi uncias duas.
Detur usui. Signetur: " Two tablespoonfuls to be taken every hour."

This he alternates with the powder of the male fern, as indicated below:

℞ Pulveris radicis filicis maris drachmas tres. Divide in doses numero tres.
Signetur: " One powder stirred up in tea to be taken every hour alternately with the oil."

These measures, we are informed, tend to the fulfilment of the first intention, that of effecting the destruction of the worm. The actual removal of the tænia, dead or alive, is aimed at by the exhibition of calomel in union with gamboge. The author never, but with the greatest reluctance, substitutes any other drastic, however similar in its operation, as none besides, in his own estimation, act so beneficially or so specifically. The dose is regulated according to the particular diathesis of the patient; but, generally speaking, and on an average, the remedy is taken according to the following formula:

℞ Calomelanos.
Pulveris Gummi guttæ [Cambogiæ.]
Sacchari Albi aa grana sex.

Exacte terendo fiat pulvis. Deutur tales doses numero tres. Signetur; "A powder to be taken at one, three, and five in the afternoon."

To provide against any possible nausea or disgust at these remedies, the author is in the habit of being provided with some ounces of candied orange-peel to serve as a means of clearing the mouth by mastication, and also with some infusion of lime-tree flowers, to be drunk after the medicines have been taken. It is also advisable to have some emollient fluids in readiness, in case of clysters or fomentations being required for any purpose.

These various means require the strictest regularity and order in their management; and the precise mode prescribed of administering the remedies must be conscientiously adhered to. In support of this injunction, Dr. Wawruch appeals to the recognized practical truth which demands that the physician, in employment of any specific mode of cure, should observe a rigorous punctuality, even in things apparently the most trifling. In particular, the succession of doses should receive the most careful attention; for, if these be given too precipitately, they will assuredly be rejected by vomiting, and the object be frustrated. If the intervals be too protracted, the patient becomes depressed and impatient, and, without purpose, suffers longer than he ought.

To resume; our author commences with the final treatment at 8 a.m. by administering the first dose of castor-oil; at 8½ a.m. the male fern is taken; at 9 a.m. the second dose of castor-oil is received, and in half an hour the next powder of the filix mas. At 10 a.m. the third and last dose of oil is given, and at 10½ the last of the powders; in the hour next ensuing, one or two injections of milk and oil are administered. From this time till about 1 p.m., repose in every respect is conceded to the patient, in order that the medicines received should duly attain the intestinal surface, and be able to exert their influence upon the tapeworm itself.

At this period, nausea, headach, and griping most commonly set in, in such a manner that the patient will often conceive, from the intestinal commotion, that he can point out the precise location of the parasite. If vomiting now arise, the entire process is procrastinated, as in that case the whole of the third dose, or at least one half of the male fern powder must be repeated. If this interval, however, pass without the occurrence of any material accident, the practitioner must proceed to the administration of the gamboge and calomel, selecting, for fear of vomiting, the moment of greatest tranquillity on the part of the patient. It is well to let him have some candied orange-peel just after the dose has been taken, as this often settles the medicine on the stomach. Time must be allowed before a second exhibition of the drastic; if nothing result for an hour or an hour and a half, the next dose may be given. Most commonly, however, if the medicine do not disagree, intestinal action will speedily take place after its administration; and the patient, with violent spasmodic action of the sphincter ani, expels the tapeworm. Of course, after this event, no further drugs are given, even though the head of the parasite be not discovered, as the cure is not the less certain on this account. Soon after the expulsion, all inconvenience to the patient ceases, who feels thoroughly refreshed, and as one regenerated.

The above description of treatment, with its results, is drawn from the more fortunate cases, a period of six hours at the most, being generally consumed in its progress. At times, however, the occurrence of fever, or of undue nervous excitement, during the preliminary abstinence, will necessitate the postponement of the ultimate remedies; at others, an indomitable vomiting has seemed to arise from the bulk of the male fern, which may equally frustrate the intention of the physician. Dr. Wawruch does not approve of the exhibition, in these cases, of the *filicin* as a substitute, according to the suggestion of the Baron von Jacquin, as, in three instances in which he had given this alkaloid, most severe and troublesome symptoms followed its use. The castor-oil also, and the drastic of gamboge and calomel, will occasionally so excite the stomach and bowels, as to render the practitioner's course both difficult and doubtful; and, here, acting upon general principles, he must be guided by judgment and discretion.

Another embarrassing circumstance consists in the expulsion of the worm not taking place somewhere about the customary period, since it creates a doubt whether the drastics should be withdrawn, or be continued to an unwonted degree. In these cases, the author's experience leads him to wait, and generally in a day or two, probably after the idea of cure has been given up, the wished-for result occurs without any medicine being given anew.

Whether an attempt, as here described, at the effective cure of tænia be successful or otherwise, the after-treatment must be decidedly such as shall tend to obviate the fear of intestinal inflammation, a state of things to be guarded against on account of the excitability induced by the previous proceedings. Our author, in this case, directs the treatment commonly recommended and employed in ordinary irritation of the gastro-intestinal mucous membrane. Some hours after the expulsion of the tapeworm, occasionally in a few minutes, a violent hunger sometimes arises, which must be carefully attended to, as the most prejudicial consequences have been seen to follow the indulgence at this period of the inclination. At times, however, no sense of inquietude, or irregularity of any kind, succeeds the treatment; in these cases, it is desirable not the less to warn the patient to be careful respecting diet for at least a few days.

We now conclude our analysis of this very interesting and valuable monograph. Before closing this article, we may repeat what was stated at the onset, that the doctrines inculcated are illustrated by an appendix containing accounts of 206 cases. These are concisely related, the main features of each being given without that troublesome repetition and detail which often characterize clinical histories. We have looked here and there into the appendix, without, however, having systematically gone through with it; and the published results, so far as we can judge, would seem to coroborrate the estimate which Dr. Wawruch attaches to his own plan. As stated in the title-page, the work is prefaced by Professor Bischoff, in terms highly laudatory of its contents and its author; and, certainly, as a contribution to practical medicine, its commendation is only an act of justice. We suspect, however, that the plan of treatment will hardly ever be likely to become general, at any rate out of the wards of an hospital. Many cases will undoubtedly be cured by a much more simple, and, we may add, less perilous process; and then again, the difficulty of accomplishing the plan in all its integrity would be found insufferably great in a majority of instances. For our own parts, we feel disposed to give the method a trial, in cases that shall resist and baffle common treatment, especially where the patients' anxiety or intelligence would furnish a reasonable assurance of his own cooperation.

To such of our readers as are familiar with the German language, we strongly recommend a perusal of the book itself; it is written in a pleasing and even elegant style it abounds; with anecdote and lively illustration; and it is well calculated in this way, as well as by its practical worth, to recommend its doctrines to the favorable attention of the profession.

---

### Art. V.

*Guy's Hospital Reports. Second Series.* Nos. I, II, and III. April, 1843, to April, 1844. *London,* 8vo.

